# Clinical Patterns in Patients with Basal Cell Carcinoma: A 10-Year Single-Center Retrospective Study

**DOI:** 10.3390/medicina62091696

**Published:** 2026-09-04

**Authors:** Eliza Rebeka Siemaszko-Oniszczuk, Przemysław Hałubiec, Anna Wojas-Pelc, Andrzej Kazimierz Jaworek

**Affiliations:** 1 Department of Dermatology and Allergology, University Hospital, Botaniczna 3, 31-503 Krakow, Poland; esiemaszko@su.krakow.pl (E.R.S.-O.); anna.wojas-pelc@uj.edu.pl (A.W.-P.); andrzej.jaworek@uj.edu.pl (A.K.J.); 2 Department of Dermatology, Jagiellonian University Medical College, Botaniczna 3, 31-503 Krakow, Poland; 3 Doctoral School of Medical and Health Sciences, Jagiellonian University Medical College, Łazarza 16, 31-530 Krakow, Poland

**Keywords:** basal cell carcinoma, recurrence, H-zone, high-risk histology, epidemiology

## Abstract

*Background and Objectives*: Basal cell carcinoma (BCC) is the most common non-melanoma skin cancer, and its global incidence rate constantly rises. However, there are no current epidemiological data for many regions, including Małopolska in southern Poland. This study aimed to characterize the clinical and histological profile of patients with BCC and to identify factors associated with local recurrence and the presence of multiple tumors. *Materials and Methods*: We performed a single-center, retrospective observational study consistent with STROBE guidelines at the Department of Dermatology and Allergology, University Hospital in Cracow. The included patients were adults with at least one histologically confirmed BCC treated between 2015 and 2025. Demographic, clinical, histopathological, and follow-up data were collected at patient and lesion levels. The results were evaluated using univariable tests, multivariable logistic regression, Kaplan–Meier survival analysis, and generalized estimating equations. *Results*: We included 108 patients (median age at first diagnosis, 73 years; 52% male) with 418 BCCs; the median follow-up duration was 84 months. Superficial BCC was the most common subtype among lesions with available histological subtype information (56%). The head and neck region was the most frequent anatomical site in this group (51%). Multiple BCCs were present in 62% of patients. Longer follow-up was independently associated with the presence of multiple BCCs. A history of actinic keratoses showed a positive but statistically nonsignificant association with multiple BCCs. Recurrence was observed in 15 lesions (3.6%). Female sex and H-zone involvement showed higher odds of recurrence. *Conclusions*: In this elderly cohort, multiple BCC tumors may reflect longer follow-up and cumulative actinic damage. Recurrence was relatively infrequent but associated with clinically relevant features, including H-zone involvement and female sex. These findings support an individualized, multifactorial approach to treatment and follow-up, taking into account age, sex, lesion burden, anatomical location, and histological subtype.

## 1. Introduction

Non-melanoma skin cancers (NMSCs), which primarily include basal cell carcinoma (BCC) and squamous cell carcinoma (SCC), are the most common malignant tumors in humans [1]. Approximately 80% of NMSCs are BCCs and 20% are SCCs, although this difference narrows with population ageing [2]. The age-standardized global incidence of NMSC continues to increase, with the highest rates observed in Australasia and North America [3]. The lifetime risk of developing at least one BCC has been estimated at approximately 30% in fair-skinned populations. Due to its generally low mortality compared with many other malignancies, the impact of BCC on patients’ lives and public health may be underestimated [4]. Nonetheless, the burden of skin cancer is projected to further increase in ageing populations [5] and its treatment is expected to impose increasing costs on healthcare systems [6].

The classic clinical picture is a solitary lesion that might present as a nodule or an erythematous patch and usually grows slowly, sometimes accompanied by erosion or ulceration. BCC can arise on virtually any hair-bearing skin, although it occurs most commonly on sun-exposed sites [2]. The disease course is slow and individual lesions might grow for months or even years without causing substantial morbidity. BCC is characterized by locally invasive growth and an exceptionally low risk of distant metastasis [2,7]. Despite this, if left untreated, BCC may progressively enlarge and infiltrate surrounding tissues. Ultimately, lesions might become very large, ulcerated, and disfiguring, at which stage treatment becomes very challenging.

Therefore, early diagnosis remains important to prevent such unfavorable consequences. Although many BCCs grow with recognizable clinical features, presenting as a chronic lesion with a raised or infiltrated border, adjunctive tools may facilitate diagnosis. There are multiple characteristic dermoscopic features, including arborizing vessels, shiny white lines or structures visible under polarized light, and pigmented structures such as blue-gray ovoid nests, leaf-like areas, and spoke-wheel structures [8]. In case of doubt or when histopathological confirmation is required for definitive treatment planning, a diagnostic biopsy is performed [7].

Treatment of BCC remains largely surgical, with complete surgical excision considered the first-line treatment, allowing histopathological assessment of resection margins [7]. However, some patients may decline surgery, may not be suitable surgical candidates, or may have multiple tumors for which extensive surgical management could result in substantial morbidity. In appropriately selected, usually low-risk lesions, non-surgical methods such as cryotherapy, photodynamic therapy, laser ablation, or topical imiquimod may be considered [4,9].

Despite its generally favorable prognosis, BCC is clinically and biologically heterogeneous. It comprises several histological subtypes ranging from indolent superficial and nodular forms to infiltrative, micronodular, morpheaform, and basosquamous types associated with a higher risk of incomplete excision, local recurrence and tissue destruction [7].

A significant proportion of patients develop more than one BCC over their lifetime. Recurrences and multiplicity contribute to higher morbidity, treatment burden and healthcare costs associated with this otherwise low-mortality cancer. Characterizing these clinically relevant outcomes is important to understand the real-world burden of BCC in a given population.

In the Polish setting, the current epidemiological profile of patients with BCC is insufficiently recognized. Although valuable data from some Polish centers are available [10], no up-to-date comprehensive analysis of this patient population has been conducted in the Małopolska region.

This gap in knowledge is important because BCC incidence, distribution of subtypes and anatomical locations vary geographically with latitude, UV exposure and phototype of the local population with the additional contribution of demographic structure and healthcare-seeking behaviors [2]. Consequently, epidemiological estimates from distinct regions may differ from generalized or pooled estimates.

Cracow lies at approximately 50° N latitude in a temperate continental climate, placing it within the moderate UV-exposed area of Central Europe as opposed to the high-UV places (e.g., Australasia), which are associated with the highest BCC incidence [2]. The regional population is predominantly Caucasian, with fair-to-intermediate skin pigmentation (Fitzpatrick phototypes I-III), which itself is a risk factor for BCC. Occupational UV exposure, including agricultural work, may be an additional risk factor for BCC, as suggested by observations among Polish farmers [11].

The aim of this study was to characterize the clinical patterns among patients with BCC, with particular emphasis on clinically relevant outcomes such as recurrence and the presence of multiple BCCs.

## 2. Materials and Methods

This single-center retrospective observational study was conducted at the Department of Dermatology and Allergology, University Hospital in Krakow. The study was reported in accordance with the Strengthening the Reporting of Observational Studies in Epidemiology (STROBE) statement [12]. Data were extracted from hospital medical records. All patients managed between 2015 and 2025 who fulfilled the inclusion criteria were considered eligible.

The inclusion criteria were (1) at least one histopathologically confirmed BCC; and (2) age ≥ 18 years.

Collected data included demographic, clinical, histopathological, diagnostic, therapeutic, and follow-up information. In some cases, full histopathological reports were unavailable. These lesions were excluded from the main lesion-specific analysis.

Recurrence was defined as histologically documented reappearance of BCC at the previously treated site during follow-up. The facial H-zone was defined as the central face (including the eyelids, eyebrows, periorbital region, nose, upper lip), jaw angles, temples, ears, and pre- and postauricular regions [13]. High-risk histology was defined as the presence of infiltrative, micronodular, morpheaform, or basosquamous BCC [7,14].

All patients were managed according to standard clinical practice and in accordance with Polish and European guidelines [7,14]. Management decisions were made at the discretion of the treating dermatologist, based on patient characteristics and preferences, and tumor features.

The study was conducted in accordance with the 2024 revision of the Declaration of Helsinki [15] and was approved by the Research Ethics Committee at the Jagiellonian University Medical College (approval no. 1072.6120.2.249.2026, issued on 10 June 2026). Given the retrospective nature of the medical record-based analysis, the requirement for informed consent was waived by the Ethics Committee.

### Statistical Analysis

Numerical variables were summarized as medians with interquartile ranges (IQRs), and categorical variables as counts and percentages. Comparisons between groups were done using the Wilcoxon rank-sum test for numerical variables and Fisher’s exact test for categorical variables. The χ^2^ test with Monte Carlo simulation was used to evaluate associations between multiple categorical groups.

The multivariable logistic regression models were constructed using a preselected set of variables based on their clinical relevance as well as the structure of the available data. Population-averaged logistic regression models fitted with generalized estimating equations and an exchangeable working correlation structure were used to account for clustering of multiple lesions within individual patients.

Recurrence-free survival was estimated using the Kaplan–Meier method. The log-rank test was used to evaluate the relationship between recurrence-free survival and specific variables.

Missing values were not imputed. There was no adjustment for multiple comparisons because of the exploratory nature of the study. To assess potential selection bias related to missing histological subtype data, lesions with and without available subtype information were compared. Sensitivity analyses were conducted after excluding patients with more than 10 recorded BCCs. All tests were two-sided, and *p*-values < 0.05 were considered statistically significant. All analyses were performed using R version 4.6.0 (R Foundation for Statistical Computing, Vienna, Austria) [16].

## 3. Results

**Figure 1:**
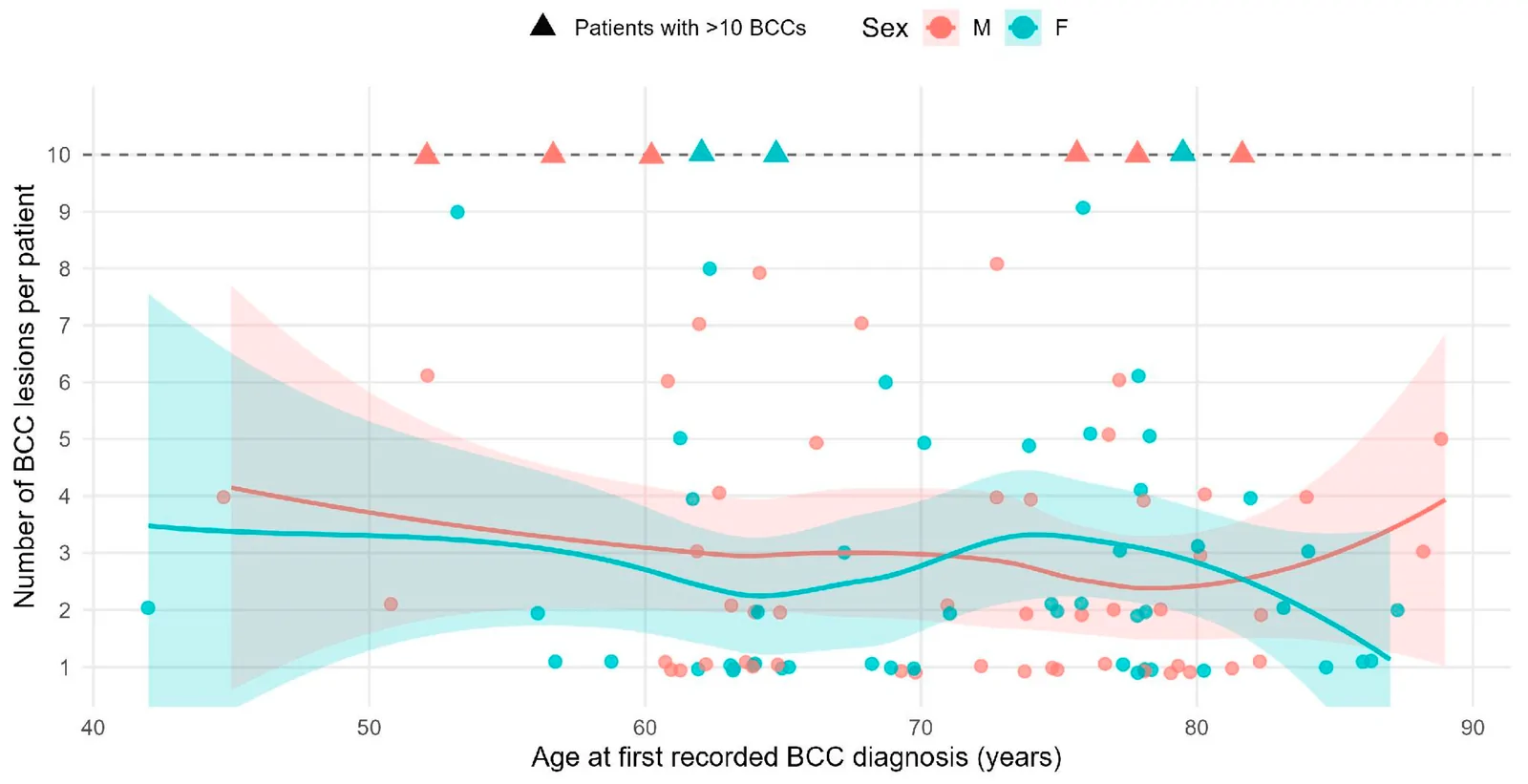
Number of BCC lesions per patient according to age at the first recorded BCC diagnosis and sex. Patients with more than 10 BCCs are projected onto the cutoff line at 10 lesions. Abbreviations: BCC—basal cell carcinoma.

A total of 108 adult patients with 418 recorded BCC lesions were included in the study. The median age at the first BCC diagnosis was 73 years (IQR 63–78), and 56 patients (52%) were male (Figure 1). Median follow-up duration was 84 months (IQR 48–120). Multiple BCCs were recorded in 67 patients (62%). A history of actinic keratosis (AK) was present in 52 patients (48%), squamous cell carcinoma (SCC) in 13 (12%), SCC in situ/Bowen disease in 11 (10%), and malignant melanoma in 12 (11%). Hypertension was the most common comorbidity, present in 45 patients (42%). Patient characteristics are presented in Table 1.

Compared with patients with a single BCC, those with multiple BCCs had a longer follow-up duration (median 102 months, IQR 51–120, versus 60 months, IQR 36–96; *p* = 0.013) and were more likely to have a history of AK (59.7% versus 29.3%; *p* = 0.003). No other statistically significant between-group differences were observed.

In the multivariable logistic regression model, longer follow-up was associated with higher odds of having multiple BCCs (OR per year = 1.18, 95% CI 1.03–1.37; *p* = 0.021). A history of AK showed a positive but statistically nonsignificant association (OR = 2.64, 95% CI 0.94–7.45; *p* = 0.066) (Table 2).

A histological subtype was available for 261 BCC lesions (62.4%) in 68 patients. Among these, superficial BCC was the most frequent subtype (56%), followed by nodular BCC (28%). Overall, 40 lesions (15%) were classified as having high-risk histology. The head and neck region was the most common anatomical site (51%), followed by the trunk (28%). H-zone involvement was recorded in 19% of lesions with available location data. Surgery was the most common treatment (88%).

Detailed lesion characteristics are presented in Table 3. The distributions of the examined characteristics were similar between lesions with and without available histological subtype information, although lesions without subtype information had longer follow-up (Table S1).

**Figure 2:**
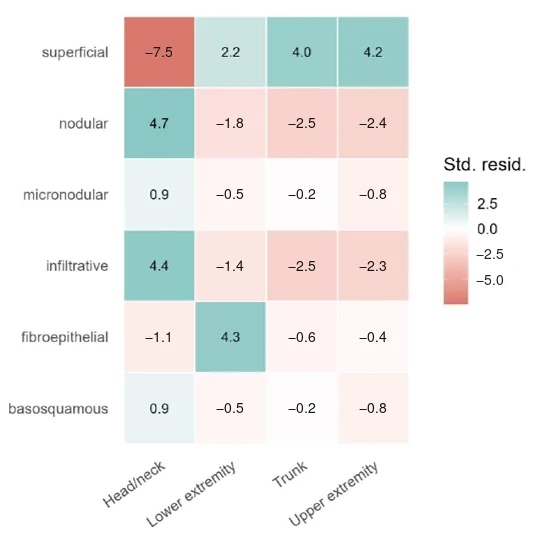
Association between BCC histological subtype and anatomical region. The heatmap presents adjusted standardized residuals from the analysis of BCC subtype by anatomical region.

Histological subtype was significantly associated with anatomical region (χ^2^ = 80.53; Monte Carlo *p* < 0.001; Cramér’s V = 0.326; Figure 2). Nodular and infiltrative BCCs were disproportionately located in the head and neck region, whereas superficial BCCs were more frequently located on the trunk and extremities. Non-surgical treatment was used for 31 superficial BCCs (21.2%), compared with one BCC of another histological subtype (0.9%; Fisher’s exact test, *p* < 0.001).

Overall, recurrence was documented in 15 BCC lesions (3.6%). Among lesions with an available histological subtype, recurrence was documented in 10 lesions (3.8%). All 10 recurrent lesions in this analytical subset were located in the head and neck region and had initially been treated surgically. The median time to recurrence was 18 months (IQR 12–54 months).

In univariable lesion-level GEE models, female sex was associated with higher odds of recurrence (OR = 3.55, 95% CI 1.05–12.01; *p* = 0.042), as was H-zone involvement (OR = 4.84, 95% CI 1.21–19.43; *p* = 0.026). High-risk histology showed an association in the same direction but did not reach statistical significance (OR = 4.07, 95% CI 0.89–18.52; *p* = 0.070) (Table 4). A multivariable model was not fitted because of the low number of recurrence events.

Kaplan–Meier analysis demonstrated lower recurrence-free survival among lesions with high-risk histology than among those with low-risk histology (log-rank *p* = 0.035). A similar borderline-significant difference was observed for H-zone involvement (log-rank *p* = 0.056).

Sensitivity analyses excluding patients with more than 10 recorded BCCs produced estimates in the same direction as the primary analyses, although with reduced statistical precision (Tables S2 and S3).

The non-significant results of exploratory analyses that were not presented above are provided in the Supplementary Material (Table S4).

## 4. Discussion

Our study characterizes adult patients from a single center in the Małopolska region of Poland who had at least one histopathologically confirmed BCC. The cohort consisted predominantly of older individuals, and the head and neck region was the most commonly affected site. Superficial BCC accounted for approximately half of the tumors with an available histological subtype, and surgery was by far the most commonly used treatment modality.

These observations are consistent with the previous reports on the epidemiology of BCC [10,17,18]. The high frequency of head and neck involvement in our study is consistent with data indicating a particularly high burden of BCC at chronically sun-exposed sites [19]. Ultraviolet radiation (UV), especially ultraviolet B (UVB), is the primary environmental risk factor for BCC, with both cumulative and intermittent exposure contributing to tumor development [20]. Comprehensive photoprotection, including seeking shade, wearing protective clothing, and using sunscreen, remains a key preventive strategy against NMSCs, including BCC [21,22].

Regional comparisons provide additional context for the clinical patterns observed in our cohort. In another Polish study, nodular BCC was the predominant histological subtype (44.0%), followed by superficial (30.5%) and infiltrative BCC (14.1%). In that study, 73% of BCCs were located on the face [10]. Swedish registry data showed an anatomical distribution more similar to that observed in our cohort, with 54% of BCCs located on the head and neck and 31% on the trunk. However, the histological distribution again differed, as nodular, superficial, and combined micronodular/infiltrative BCCs accounted for 31%, 19%, and 31% of registered tumors, respectively [17]. Interestingly, in tropical North Queensland, superficial BCC occurred at higher rates than previously reported in less sun-exposed populations and showed a more even distribution across the face, trunk, and limbs [23]. These distributions support the hypothesis that patterns of UV exposure, together with site-specific tissue characteristics, contribute to the development of particular BCC subtypes [24,25]. Although BCC burden is naturally associated with ambient UV radiation, it must be noted that population age structure and phototype distribution, socioeconomic factors, access to dermatological care, and cancer registration practices may substantially influence actual reporting [26,27].

Differences in local tissue architecture and embryologically distinct facial sites may also explain the preferential distribution of particular subtypes [28,29]. These observations support the hypothesis that superficial and nodular BCCs arise through partially different etiological pathways [23,28].

The predominance of superficial BCC among tumors with a documented histological subtype in our elderly cohort is notable. Superficial BCC is typically characterized by shallow and sometimes multifocal growth, with a predilection for the trunk, although nodular BCC is generally regarded as the most common histological subtype [28,30]. Enhanced dermoscopic surveillance among patients with a previous diagnosis of BCC may have facilitated the detection of clinically subtle superficial BCCs that would otherwise have remained unnoticed by patients and potentially undetected during routine clinical examinations by non-dermatologists, thereby contributing to the relatively high proportion of superficial tumors in our cohort.

Superficial BCCs were more frequently treated with non-surgical modalities, consistent with established BCC treatment recommendations [13].

One of the most important findings of our study was the high prevalence of multiple BCCs. Approximately two-thirds of patients had two or more BCCs recorded. In a large Polish multicenter cohort, approximately one-fifth of patients with an initial BCC developed a subsequent BCC [31]. Similarly, approximately one-quarter of patients in a nationwide Dutch registry developed at least one subsequent BCC within 3 years [32]. In a prospective population-based study conducted in North Queensland, approximately one-quarter of patients with BCC were treated for multiple BCCs during a 3-year period [33], whereas 39.9% of patients in the Swedish BCC Registry had at least two registered tumors [34]. The longer follow-up in our cohort is the most plausible explanation for the higher proportion of patients diagnosed with multiple BCCs. This underscores the substantial cumulative clinical burden of BCC and the importance of long-term surveillance of such patients.

Among the examined factors, a history of AK was more frequent among patients with multiple BCCs, although this association did not retain statistical significance in the multivariable model. The relationship between AK and multiple BCCs supports the concept of field cancerization, in which chronically sun-damaged areas contain widespread carcinogenic alterations that predispose to the development of multiple keratinocyte cancers [35,36]. The presence of AK may therefore serve as a clinical marker of cumulative UV-induced damage and an increased risk of NMSCs [37,38].

Despite the high burden of multiple tumors, the overall proportion of recurrent lesions was relatively low and similar to the 3.3% proportion of recurrent BCCs reported in a tertiary cohort from northern Poland [39] and the 3% recurrence rate reported after surgical excision of facial BCC in an Australian center [40].

H-zone involvement was associated with higher odds of recurrence. This finding is consistent with prior studies identifying facial high-risk zones and periorificial areas as sites associated with an increased risk of recurrence [38,40,41]. Several factors may explain this association, including the use of narrower surgical margins in cosmetically and functionally sensitive areas, together with complex anatomical boundaries [40]. Female sex was also associated with higher odds of recurrence. This contrasts with studies reporting a higher burden of BCC among older men as well as an association between male sex and the occurrence of multiple BCCs [42,43]. Although sex-specific differences in genetic susceptibility to BCC have been described, these studies primarily concern the risk of developing BCC rather than the risk of post-treatment recurrence [44].

Our findings support a multifactorial approach to risk stratification and management of BCC in older patients. Ultimately, treatment decisions should integrate patient-related factors, including age, comorbidities, functional status, and preferences, with tumor-related characteristics such as lesion size, anatomical location, histological subtype, and recurrent status [7,13,14].

Patients with high-risk features, such as aggressive histological subtypes, larger tumor size, or a history of previous or recurrent BCC, may require particular attention during surgical planning, margin assessment, and long-term surveillance [45,46]. For appropriately selected high-risk or recurrent tumors, Mohs micrographic surgery or alternative margin-controlled techniques, including staged excision with paraffin-embedded margin assessment and complete-margin intraoperative frozen-section excision, may reduce recurrence while preserving uninvolved tissue and optimizing functional and cosmetic outcomes [7,13,47]. Preoperative imaging may further support surgical planning in these settings. For example, 20 MHz high-frequency ultrasound combined with a depth-correction equation has been shown to estimate histopathologic BCC depth within clinically acceptable limits [48]. Other noninvasive imaging techniques, including optical coherence tomography (OCT), line-field confocal OCT and reflectance confocal microscopy, have also been used for presurgical margin and depth assessment and may reduce the number of surgical stages required for complete tumor excision [49]. These approaches could be particularly valuable for tumors in the functionally and cosmetically important, high-risk areas identified in the present study as more prone to recurrence. In addition, locally advanced or aggressive tumors may require multidisciplinary assessment and additional local or systemic treatment.

Some of the challenges encountered in the present study also highlight broader practical issues associated with BCC in clinical care and research. Because the diagnosis and management of most BCCs are relatively straightforward in routine clinical practice, detailed lesion-level information may not always be systematically recorded. Greater standardization of BCC documentation could improve both clinical management and the quality of real-world evidence. Structured or semi-automated electronic forms integrating clinical, dermoscopic, histopathological, and treatment data could facilitate consistent reporting as well as better linkage of multiple tumors developing in the same patient. This would complement efforts to improve registration of BCC and other NMSCs, which remain incompletely captured in many cancer registries [10,50,51].

Several aspects of the diagnostic and therapeutic approaches also remain suitable for further evaluation. The role of pretreatment biopsy should be considered in the context of the individual lesion, as prior knowledge of an aggressive histological subtype or other high-risk features may influence risk stratification, treatment selection, surgical planning, and margin assessment [7,13,14,45,46]. More standardized histopathological reporting of clinically relevant prognostic features could further improve comparability between institutions and facilitate future multicenter studies.

At the same time, increasing BCC incidence in older populations raises the opposite problem of potential overtreatment. In patients with substantial comorbidity, limited life expectancy, or high overall medical burden, the expected benefit of invasive treatment of every low-risk lesion (particularly some cases of superficial BCC) should be carefully weighed against treatment burden, functional outcomes, and patient preferences [7,13,14]. Such decisions are particularly challenging in the context of high numbers of tumors observed in some patients [31,32,33,34]. Resolution of these issues should be a major focus of future research on BCC.

This study has several limitations. The retrospective single-center design may limit the generalizability of the findings, and the results depend on the quality and completeness of the original medical records. Exact dates of diagnosis were unavailable or could not be reliably established for a considerable proportion of individual lesions. Consequently, tumors could not be reliably classified as synchronous or metachronous, and time to the development of a subsequent primary BCC could not be analyzed. Standardized dermoscopic findings were not consistently documented in the medical records and, therefore, could not be reliably included in the analyses. Many additional contextual factors, including individual-level phototype or occupation, could not be reliably obtained from the medical records. Detailed histopathological reports, including information on BCC subtype, were unavailable for some lesions. Analyses involving histological subtype were restricted by data availability and may have been affected by selection or information bias. Consequently, several established recurrence-related factors such as tumor size, surgical margin width or perineural invasion could not be reliably included in the analyses. Their absence limited assessment of determinants of BCC recurrence. Finally, longer follow-up increases the opportunity to identify additional primary BCCs, complicating the relationship between biological susceptibility and surveillance-related detection of subsequent tumors.

## 5. Conclusions

Our study highlights the substantial burden of patients with BCC, including subsequent BCCs and other skin tumors. The presented findings support individualized risk assessment and long-term surveillance, with particular emphasis on the careful follow-up of tumors located in high-risk anatomical sites and the recognition of extensive actinic damage.

Improved registration of NMSC, preferably including lesion-level data and subsequent primary tumors, is important because BCC remains incompletely captured by many cancer registries [10,50,51]. Integrating prevention and patient education with long-term surveillance may improve clinical outcomes and facilitate more efficient allocation of healthcare resources.

Further multicenter studies are required to validate the observed associations and determine their applicability to broader populations.

## Figures and Tables

**Table 1:** Characteristics of patients in the study cohort.

Characteristic	All Patients (N = 108)	Patients with Multiple BCCs (N = 67)
Age at first BCC diagnosis (years)	73 (63, 78)	74 (63, 78)
Sex		
Male	56 (52%)	36 (54%)
Female	52 (48%)	31 (46%)
Common cardiovascular and metabolic disorders		
Hypertension	45 (42%)	27 (40%)
History of myocardial infarction	6 (6%)	5 (8%)
Vascular disease	19 (18%)	13 (19%)
Diabetes mellitus	16 (15%)	9 (13%)
Smoking	8 (7%)	5 (8%)
Alcohol use	5 (5%)	4 (6%)
Selected medications		
ASA	16 (15%)	10 (15%)
Statins	25 (23%)	15 (22%)
Immunosuppressive medications	9 (8%)	4 (6%)
History of non-cutaneous cancer	10 (9%)	8 (12%)
History of AK	52 (48%)	40 (60%)
History of SCC	13 (12%)	10 (15%)
History of SCCis/Bowen disease	11 (10%)	9 (13%)
History of MM	12 (11%)	10 (15%)

Data are presented as median (IQR) or n (%). Abbreviations: AK—actinic keratosis; ASA—acetylsalicylic acid; BCC—basal cell carcinoma; MM—malignant melanoma; SCC—squamous cell carcinoma; SCCis—squamous cell carcinoma in situ.

**Table 2:** Multivariable logistic regression model for the presence of multiple BCCs.

Variable	OR	95% CI	*p*-Value
Age at first recorded BCC diagnosis, per 10 years	1.08	0.66–1.78	0.756
Female sex	0.62	0.22–1.73	0.362
Follow-up duration, per year	1.18	1.03–1.37	0.021
History of actinic keratosis	2.64	0.94–7.45	0.066

Abbreviations: BCC—basal cell carcinoma; CI—confidence interval; OR—odds ratio.

**Table 3:** Characteristics of BCC lesions with available histological subtype information.

Characteristic	BCC Lesions (N = 261)
BCC subtype	
nodular	74 (28%)
superficial	146 (56%)
micronodular	4 (1.5%)
infiltrative	32 (12%)
basosquamous	4 (1.5%)
fibroepithelial	1 (0.4%)
morpheaform	0 (0%)
Location	
head and neck	134 (51%)
trunk	72 (28%)
upper extremity	34 (13%)
lower extremity	13 (5%)
missing data	8
H-zone	48 (19%)
Management	
surgical	226 (88%)
cryosurgery	20 (8%)
PDT	7 (2.7%)
imiquimod	1 (0.4%)
5-FU	2 (0.8%)
radiotherapy	2 (0.8%)
missing data	3
Follow-up (months)	36 (24, 78)

Data are presented as median (IQR) or n (%). Abbreviations: BCC—basal cell carcinoma; 5-FU—5-fluorouracil; PDT—photodynamic therapy.

**Table 4:** Univariable lesion-level GEE logistic regression models for BCC recurrence.

Variable	OR	95% CI	*p*-Value
Age at BCC diagnosis, per 10 years	1.31	0.81–2.10	0.269
Female sex	3.55	1.05–12.01	0.042
High-risk histology	4.07	0.89–18.52	0.070
H-zone involvement	4.84	1.21–19.43	0.026

Abbreviations: BCC—basal cell carcinoma; CI—confidence interval; GEE—generalized estimating equations; OR—odds ratio.

## Data Availability

The original contributions presented in this study are included in the Supplementary Material. Further inquiries can be directed to the corresponding author.

## Supplementary Materials

The following supporting information can be downloaded at: https://www.mdpi.com/article/10.3390/medicina62091696/s1, Table S1: Comparison of BCC lesions with and without available histological subtype information; Table S2: Comparison of patients retained in and excluded from the sensitivity analysis after exclusion of patients with >10 recorded BCCs; Table S3: Comparison of regression estimates in the primary and sensitivity analyses after exclusion of patients with >10 recorded BCCs. Table S4. Non-significant results from exploratory analyses. Dataset: File containing raw data supporting the findings of the study.

## Abbreviations

The following abbreviations are used in this manuscript:

5-FU	5-fluorouracil
AK	actinic keratosis
ASA	acetylsalicylic acid
BCC	basal cell carcinoma
CI	confidence interval
GEE	generalized estimating equations
IQR	interquartile range
MM	malignant melanoma
NMSC	non-melanoma skin cancer
OCT	optical coherence tomography
OR	odds ratio
PDT	photodynamic therapy
SCC	squamous cell carcinoma
SCCis	squamous cell carcinoma in situ
STROBE	Strengthening the Reporting of Observational Studies in Epidemiology
UV(B)	Ultraviolet (B)
