# Supplementary material for: Clinical Patterns in Patients with Basal Cell Carcinoma: A 10-Year Single-Center Retrospective Study
Source: Medicina (Kaunas). 2026 Sep 4;62(9):1696. doi: 10.3390/medicina62091696 (PMC13609343; doi:10.3390/medicina62091696)
Supplement: Supplementary file 1 [file medicina-62-01696-s001.zip › Supplementary file.pdf]

**Table S1.** Comparison of BCC lesions with and without available histological subtype information.

| Characteristic | Histological subtype unavailable N = 157 | Histological subtype available N = 261 |
|---|---|---|
| Age at BCC diagnosis (years) | 75 (65, 80) | 72 (64, 80) |
| Sex | | |
| Male | 83 (53%) | 156 (60%) |
| Female | 74 (47%) | 105 (40%) |
| Anatomical region | | |
| Head and neck | 95 (61%) | 134 (51%) |
| Trunk | 39 (25%) | 72 (28%) |
| Upper extremity | 9 (6%) | 34 (13%) |
| Lower extremity | 9 (6%) | 13 (5%) |
| Not recorded | 5 | 8 |
| H-zone | 35 (22%) | 48 (19%) |
| Management | | |
| Surgical treatment | 144 (94%) | 226 (88%) |
| Cryosurgery | 6 (4%) | 20 (8%) |
| Photodynamic therapy | 3 (2%) | 7 (3%) |
| Imiquimod | 0 (0%) | 1 (0.4%) |
| 5-Fluorouracil | 1 (1%) | 2 (1%) |
| Radiotherapy | 0 (0%) | 2 (1%) |
| Recurrence | | |
| No | 152 (97%) | 251 (96%) |
| Yes | 5 (3%) | 10 (4%) |
| Follow-up duration, months | 60 (36-108) | 36 (24-78) |

Data are presented as median (interquartile range) or n (%).

**Abbreviations:** BCC—basal cell carcinoma.

**Table S2.** Comparison of patients retained in and excluded from the sensitivity analysis after exclusion of patients with >10 recorded BCCs.

| Characteristic | Retained patients, ≤10 BCCs N = 99 | Excluded patients, >10 BCCs N = 9 | p-value |
|---|---|---|---|
| Age at first recorded BCC diagnosis, years | 73 (64-78) | 64 (61-78) | 0.344 |
| Female sex | 49 (49.5%) | 3 (33.3%) | 0.492 |
| Follow-up, months | 72 (36-120) | 120 (108-126) | 0.013 |
| Any BCC recurrence | 10 (10.1%) | 3 (33.3%) | 0.075 |
| History of non-cutaneous cancer | 7 (7.1%) | 3 (33.3%) | 0.036 |
| History of actinic keratosis | 46 (46.5%) | 6 (66.7%) | 0.308 |
| History of squamous cell carcinoma | 11 (11.1%) | 2 (22.2%) | 0.296 |
| History of SCC in situ/Bowen disease | 9 (9.1%) | 2 (22.2%) | 0.228 |
| History of malignant melanoma | 8 (8.1%) | 4 (44.4%) | 0.008 |

Data are presented as median (interquartile range) or n (%).

**Abbreviations:** BCC—basal cell carcinoma; SCC—squamous cell carcinoma.

**Table S3.** Comparison of regression estimates in the primary and sensitivity analyses after exclusion of patients with >10 recorded BCCs.

| Model 1 - multivariable logistic regression for multiple BCCs | | | | |
|---|---|---|---|---|
| | Primary analysis | | Sensitivity analysis | |
| **Variable** | **OR (95% CI)** | **p-value** | **OR (95% CI)** | **p-value** |
| Age at first recorded BCC diagnosis, per 10 years | 1.08 (0.66-1.78) | 0.756 | 1.11 (0.68-1.83) | 0.676 |
| Female sex | 0.62 (0.22-1.73) | 0.362 | 0.67 (0.24-1.89) | 0.450 |
| Follow-up duration, per year | 1.18 (1.03-1.37) | 0.021 | 1.15 (0.99-1.33) | 0.059 |
| History of actinic keratosis | 2.64 (0.94-7.45) | 0.066 | 2.68 (0.95-7.52) | 0.061 |
| **Model 2 - univariable lesion-level GEE logistic regression for BCC recurrence** | | | | |
| | **Primary analysis** | | **Sensitivity analysis** | |
| **Variable** | **OR (95% CI)** | **p-value** | **OR (95% CI)** | **p-value** |
| Age at BCC diagnosis, per 10 years | 1.31 (0.81-2.10) | 0.269 | 1.28 (0.61-2.66) | 0.511 |
| Female sex | 3.55 (1.05-12.01) | 0.042 | 2.97 (0.53-16.67) | 0.217 |
| High-risk histology | 4.07 (0.89-18.52) | 0.070 | 4.46 (0.73-27.11) | 0.105 |
| H-zone involvement | 4.84 (1.21-19.43) | 0.026 | 5.10 (0.88-29.34) | 0.068 |

**Abbreviations**: BCC—basal cell carcinoma; CI—confidence interval; GEE—generalized estimating equations; OR—odds ratio.

**Table S4.** Non-significant results from exploratory analyses.

| Patient-level comparisons between patients with single and multiple BCCs | | | |
|---|---|---|---|
| **Characteristic** | **Single BCC** N = 41 | **Multiple BCCs** N = 67 | **p-value** |
| Current/last recorded age (years) | 80.0 (74.0, 88.0) | 84.0 (73.0, 88.0) | 0.927 |
| Age at first recorded BCC diagnosis (years) | 70.0 (64.0, 78.0) | 74.0 (63.0, 78.0) | 0.917 |
| Sex | | | 0.693 |
| M | 20 (49%) | 36 (54%) | |
| F | 21 (51%) | 31 (46%) | |
| Any BCC recurrence | 4 (9.8%) | 9 (13%) | 0.763 |
| Hypertension | 18 (44%) | 27 (40%) | 0.841 |
| History of myocardial infarction | 1 (2.4%) | 5 (7.5%) | 0.405 |
| Vascular disease | 6 (15%) | 13 (19%) | 0.609 |
| Diabetes mellitus | 7 (17%) | 9 (13%) | 0.592 |
| Smoking | 3 (7.3%) | 5 (7.5%) | 1.000 |
| Alcohol use | 1 (2.4%) | 4 (6.0%) | 0.648 |
| Acetylsalicylic acid | 6 (15%) | 10 (15%) | 1.000 |
| Statin use | 10 (24%) | 15 (22%) | 0.818 |
| Immunosuppressive medication | 5 (12%) | 4 (6.0%) | 0.296 |
| History of non-cutaneous cancer | 2 (4.9%) | 8 (12%) | 0.313 |
| History of squamous cell carcinoma | 3 (7.3%) | 10 (15%) | 0.362 |
| History of SCC in situ/Bowen disease | 2 (4.9%) | 9 (13%) | 0.201 |
| History of malignant melanoma | 2 (4.9%) | 10 (15%) | 0.127 |
| **Univariable GEE models for BCC recurrence** | | | |
| **Characteristic** | **OR** | **95%CI** | **p-value** |
| Age at BCC diagnosis, per 10 years | 1.31 | 0.81-2.10 | 0.269 |
| High-risk histology | 4.07 | 0.89-18.52 | 0.070 |

| | | | |
|---|---|---|---|
| Multiple-BCC status | 0.27 | 0.05-1.39 | 0.119 |
| History of actinic keratosis | 0.42 | 0.12-1.44 | 0.168 |
| History of SCC in situ/Bowen disease | 1.53 | 0.31-7.58 | 0.602 |
| History of malignant melanoma | 0.34 | 0.08-1.47 | 0.147 |
| History of squamous cell carcinoma | No recurrence | - | - |

**Associations between BCC histological subtype and selected characteristics**

| **Characteristic** | **p-value** |
|---|---|
| Age at first BCC diagnosis | 0.635 |
| Sex | 0.072 |
| Any recurrence | 0.511 |
| Multiple-BCC status | 0.827 |
| History of AK | 0.561 |
| History of SCC | 0.928 |
| History of SCCis/Bowen disease | 0.165 |
| History of MM | 0.339 |

Data are presented as median (interquartile range) or n (%), where applicable.

**Abbreviations:** AK—actinic keratosis; BCC—basal cell carcinoma; CI—confidence interval; GEE—generalized estimating equations; MM—malignant melanoma; OR—odds ratio; SCC—squamous cell carcinoma; SCCis—squamous cell carcinoma in situ.
